# High Isotropic Resolution T2 Mapping of the Lumbosacral Plexus with T2-Prepared 3D Turbo Spin Echo

**DOI:** 10.1007/s00062-017-0658-9

**Published:** 2018-01-10

**Authors:** Nico Sollmann, Dominik Weidlich, Barbara Cervantes, Elisabeth Klupp, Carl Ganter, Hendrik Kooijman, Ernst J. Rummeny, Claus Zimmer, Jan S. Kirschke, Dimitrios C. Karampinos

**Affiliations:** 1 Department of Diagnostic and Interventional Neuroradiology, Klinikum rechts der Isar, Technische Universität München, Ismaninger Str. 22, 81675 Munich, Germany; 2 Department of Neurosurgery, Klinikum rechts der Isar, Technische Universität München, Ismaninger Str. 22, 81675 Munich, Germany; 3 TUM-Neuroimaging Center, Klinikum rechts der Isar, Technische Universität München, Munich, Germany; 4 Department of Diagnostic and Interventional Radiology, Klinikum rechts der Isar, Technische Universität München, Ismaninger Str. 22, 81675 Munich, Germany; 5 Philips Healthcare, Röntgenstr. 24, 22335 Hamburg, Germany

**Keywords:** Lumbosacral plexus, Magnetic resonance neurography, Nerve roots, Reproducibility, Three-dimensional T2 mapping

## Abstract

**Purpose:**

Isotropic high-resolution three-dimensional (3D) magnetic resonance neurography (MRN) is increasingly used to depict even small and highly oblique nerves of the lumbosacral plexus (LSP). The present study introduces a T2 mapping sequence (T2-prepared 3D turbo spin echo) that is B1-insensitive and enables quantitative assessment of LSP nerves.

**Methods:**

In this study 15 healthy subjects (mean age 28.5 ± 3.8 years) underwent 3 T MRN of the LSP area three times. The T2 values were calculated offline on a voxel-by-voxel basis and measured at three segments (preganglionic, ganglionic, postganglionic) of three LSP nerves (S1, L5, L4) by two independent investigators (experienced and novice). Normative data for the different nerves were extracted and intraclass correlation coefficients (ICCs) were calculated to assess reproducibility and interobserver reliability of T2 measurements.

**Results:**

The T2 mapping showed excellent reproducibility with ICCs ranging between 0.99 (S1 preganglionic) and 0.89 (L5 postganglionic). Interobserver reliability was less robust with ICCs ranging between 0.78 (S1 preganglionic) and 0.44 (L5 postganglionic) for S1 and L5. A mean T2 value of 74.6 ± 4.7 ms was registered for preganglionic segments, 84.7 ± 4.1 ms for ganglionic and 65.4 ± 2.5 ms for postganglionic segments, respectively. There was a statistically significant variation of T2 values across the nerve (preganglionic vs ganglionic vs postganglionic) for S1, L5, and L4.

**Conclusion:**

Our approach enables isotropic high-resolution and B1-insensitive T2 mapping of LSP nerves with excellent reproducibility. It might reflect a robust and clinically useful method for future diagnostics of LSP pathologies.

**Electronic supplementary material:**

The online version of this article (10.1007/s00062-017-0658-9) contains supplementary material, which is available to authorized users.

## Introduction

Magnetic resonance neurography (MRN) has shown to be a powerful diagnostic tool that enables the localization and characterization of nerve pathologies [1]. Especially isotropic high-resolution three-dimensional (3D) MRN has been repeatedly used in recent years to depict even small and highly oblique nerves of the brachial plexus or lumbosacral plexus (LSP) by allowing reformatting in multiple planes. In this context, 3D turbo spin echo (TSE) or 3D fast spin echo (FSE) are most commonly used for achieving isotropic high-resolution T2-weighted nerve imaging in or near a plexus [2–4]. Furthermore, fat-suppressed 3D TSE imaging has recently been combined with the improved motion sensitized driven equilibrium (iMSDE) preparation to suppress vessel signal and improve plexus nerve delineation [5–7].

Most previous studies using high-resolution 3D MRN of plexus nerves have focused on qualitative assessments of T2-weighted signal alterations while quantitative investigations are still mostly lacking [2–6]. Although increasing efforts are being made to further improve image quality, evaluation of nerves solely based on qualitative imaging remains challenging. Instead, quantitative methods, such as T2 mapping, may provide an objective biomarker for monitoring nerve degeneration and regeneration; however, T2 quantification can be considerably affected by B0 and B1 inhomogeneities that are known to frequently occur in the LSP especially at 3 T. Thus, the present study combines 3D TSE imaging with an adiabatic T2 preparation in order to establish B1-insensitive high-resolution isotropic T2 mapping of LSP nerves. We evaluate the feasibility of this approach for extraction of quantitative T2 values from preselected LSP nerves, and hypothesize a high reproducibility and reliability of our newly introduced method.

## Materials and Methods

### Subjects

The study protocol was approved by the local ethics commission (registration number: 408/15S) and was carried out in accordance with the Declaration of Helsinki. Written informed consent was obtained from all subjects prior to imaging.

A total of 15 healthy subjects (10 male and 5 female volunteers, 28.5 ± 3.8 years) without any history of neurological or lumbar spinal diseases underwent MRN on a 3 T whole-body magnetic resonance scanner (Ingenia, Philips Healthcare, Best, The Netherlands). The MRN was performed three times in total in each volunteer on the same day with a short break between the single scans, including repositioning of the subject.

### Magnetic Resonance Imaging

The LSP of the subjects was scanned with a 16-channel torso coil array and the built-in-table posterior 12-channel coil array. First, a flow-suppressed (2 mm isotropic voxel size) T2-weighted 3D TSE sequence was performed to depict plexus anatomy [4]. Second, an adiabatic T2-prepared 3D TSE sequence with variable duration of the T2 preparation was applied for T2 mapping [8]. A modified B1-insensitive rotation (BIR-4) pulse was used for the T2 preparation to minimize the sensitivity to B0 and B1 inhomogeneities (supplementary material) [8]. The parameters for the T2 mapping sequence were as follows: field of view (FOV) 38 × 38 × 8 cm^3^, acquisition voxel 2 × 2 × 2 mm^3^, echo train length 80, T2 preparation durations of 20/40/60/80 ms, fat suppression spectral attenuated inversion recovery (SPAIR) and a scan duration of 6:48 min with a repetition time (TR) of 1.6 s and an effective echo time (TE) of the TSE shot of 15 ms. The flip angle train of the TSE readout was designed in order to achieve a constant signal plateau throughout 80% of the TSE shot duration for white matter [9].

### Post-Processing and Data Analysis

**Fig. 1:**
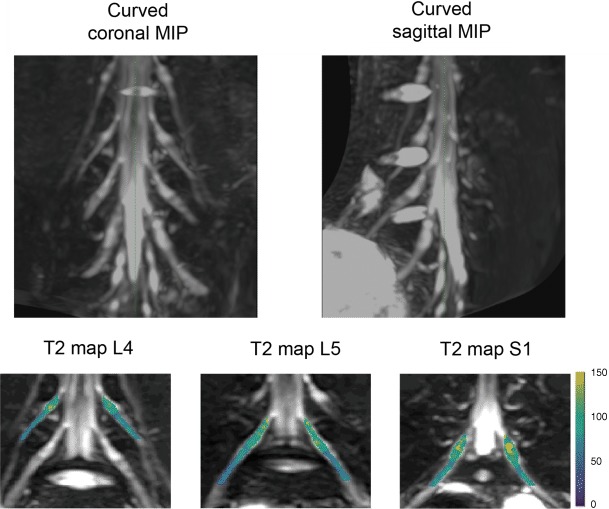
Three-dimensional (3D) turbo spin echo (TSE) imaging and T2 mapping illustrating curved coronal and sagittal maximum intensity projections (MIPs) of the lumbosacral plexus (LSP) in the *upper row*. The *bottom row* depicts color-coded coronal T2 map overlays for S1, L5, and L4

**Fig. 2:**
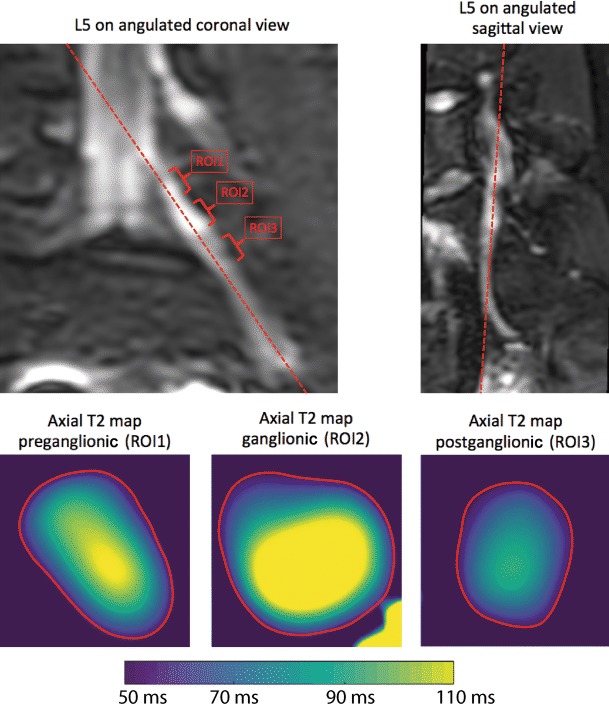
Reformatting of three-dimensional (3D) turbo spin echo (TSE) imaging and placement of regions of interest (ROIs) depicting the reformatting of imaging data in coronal and sagittal view for L5, which was conducted to display a maximum length of the nerve courses from the spinal cord to the periphery. Manual placement of ROIs was then performed separately for preganglionic (ROI1), ganglionic (ROI2), and postganglionic (ROI3) segments in axial T2 maps. T2 measurements (in ms) were then extracted for the area enclosed by the respective boundaries of the ROI (*red circles*). This was done in the same manner for preganglionic, ganglionic, and postganglionic segments of S1, L5, and L4

The T2 values were calculated offline on a voxel-by-voxel basis with a combination of variable projection (VAPRO) and golden section search [10, 11]. The resulting T2 maps were then analyzed using Horos (version 1.1.7; https://www.horosproject.org). In short, the S1, L5, and L4 nerves of both sides were identified, and a standardized color scheme was applied to the T2 maps (Fig. 1). The 3D imaging data were partly averaged (isotropic voxel size of 3 mm) and then manually reformatted to display a maximum length of the nerve course from the spinal cord to the periphery within the FOV (Fig. 2). The reformatting was done separately for S1, L5, and L4 nerves, and T2 values were measured by manually placing regions of interest (ROIs) in axial slices of the T2 maps (isotropic voxel size of 3 mm; Fig. 2). In each subject, preganglionic (~1 cm before the ganglion), ganglionic (in the middle of the ganglion), and postganglionic (~1 cm after the ganglion) T2 values were measured for each of these nerves on both sides, with uncolored anatomical images serving as reference to support precise ROI positioning based on anatomical identification of these structures (Figs. 1 and 2). To place a ROI, the respective nerve and segment were visually identified and manually surrounded using the ROI generation tool. The software then automatically displayed the mean T2 value of the area enclosed by the ROI.

The described approach of ROI positioning and measurement of T2 values was performed independently by two investigators. The first investigator (MD with experience in neuroradiological imaging since 2012: observer 1) evaluated all three scans of the subjects to assess reproducibility, whereas the second investigator (novice with experience in neuroradiological imaging since 2016: observer 2) evaluated the first scan of each subject to explore interobserver reliability. The two observers were strictly blinded to the measurements of the other, and the first observer was furthermore blinded to the T2 values of the first or first and second scan when performing measurements in the second or third scan of a respective subject. Observer 1 evaluated the first scan in each subject first, followed by a second and third round of analyses using the subjects’ second and third scans. The interval between the three analysis sessions was at least 2 weeks. Mean T2 values >100 ms were removed from the datasets prior to statistical analyses because high T2 values most likely stem from measurements in areas with high fractions of cerebrospinal fluid (CSF) or surrounding vessels.

### Statistics

For statistical data analyses, GraphPad Prism (version 6.0, GraphPad Software, La Jolla, CA) and SPSS (version 23.0, IBM, Chicago, IL) were used. The level of significance was set at *p* < 0.05 for all statistical testing.

The T2 measurements for S1, L5, and L4 of both sides extracted from the ROIs were collapsed by calculating the average of the corresponding right-sided and left-sided values (left and right S1 T2 values together, left and right L5 T2 values together, and left and right L4 T2 values together) for preganglionic, ganglionic, and postganglionic measurements, respectively. This was done separately for the values of observers 1 and 2, and descriptive statistics were calculated based on the collapsed values obtained. During this step, the portion of removed measurements due to T2 values >100 ms was documented.

Based on the mean T2 values (right-sided and left-sided T2 measurements collapsed), intraclass correlation coefficients (ICCs) were calculated to assess reproducibility and interobserver reliability (two-way mixed model, unadjusted) for the different nerves and their segments. For reproducibility, three scans in each subject, all evaluated by observer 1, were considered, whereas the first scan of each subject was evaluated independently by both observers to evaluate interobserver reliability. Furthermore, coefficients of variation (CVs) were calculated as measures for dispersion regarding the T2 values. The mean T2 values (right-sided and left-sided T2 measurements collapsed) for preganglionic, ganglionic, and postganglionic measurements of the three scans in each subject were averaged to generate one T2 value for each of these segments for S1, L5, and L4, respectively. These segment-specific values were compared by Kruskal-Wallis test followed by Dunn’s multiple comparisons test for S1, L5, and L4.

## Results

The MRN was successfully performed three times in each subject (3 × 15 = 45 scans). None of the scans had to be excluded due to artifacts according to visual image quality assessment. Observer 1 evaluated three scans in each subject with respect to preganglionic, ganglionic, and postganglionic T2 values of S1, L5, and L4 nerves of both sides (3 × 15 × 3 × 3 × 2 = 810 T2 measurements). Of these measurements, 63 (7.8%) were excluded due to T2 values >100 ms (71.4% preganglionic, 28.6% ganglionic). Furthermore, observer 2 evaluated the first scan in each subject, again separately for preganglionic, ganglionic, and postganglionic T2 values of S1, L5, and L4 nerves of both sides (1 × 15 × 3 × 3 × 2 = 270 T2 measurements). Also 32 measurements (11.9%) were removed due to T2 values >100 ms (37.5% preganglionic, 62.5% ganglionic). Mean T2 values (right-sided and left-sided T2 measurements collapsed) according to evaluation of the three scans per subject by observer 1 can be found in Table 1, whereas the mean T2 values of observer 2 are displayed in Table 2. Regarding reproducibility, excellent agreement was observed for all measurements, with ICC values ranging between 0.89 (L5 postganglionic) and 0.99 (S1 preganglionic; Table 1). The corresponding CVs ranged between 1.48% (L4 postganglionic) and 2.57% (L5 postganglionic; Table 1). Concerning interobserver reliability, good to excellent agreement was observed for all measurements of S1, whereas fair agreement was observed for L5 and poor to good agreement was present for measurements of L4 (Table 2). The interobserver ICCs ranged between 0.18 (L4 postganglionic) and 0.78 (S1 preganglionic) while the CVs ranged between 3.18% (S1 ganglionic) and 8.89% (L4 preganglionic; Table 2).

**Table 1:** Reproducibility

		Scan 1	Scan 2	Scan 3	ICC	CV (in %)
*S1* (mean T2 ± SD, in ms)	Preganglionic	74.3 ± 6.5	74.8 ± 6.7	74.4 ± 6.2	0.99	1.75
	Ganglionic	86.0 ± 5.7	86.7 ± 6.5	85.4 ± 5.9	0.98	1.64
	Postganglionic	64.5 ± 3.3	65.5 ± 3.0	65.2 ± 3.0	0.94	1.86
*L5* (mean T2 ± SD, in ms)	Preganglionic	75.1 ± 5.5	75.4 ± 5.3	76.0 ± 4.8	0.97	2.12
	Ganglionic	84.1 ± 4.0	85.1 ± 4.5	86.1 ± 5.9	0.97	1.53
	Postganglionic	66.2 ± 3.6	66.5 ± 3.3	66.8 ± 3.1	0.89	2.57
*L4* (mean T2 ± SD, in ms)	Preganglionic	72.6 ± 4.6	72.5 ± 4.5	72.6 ± 5.2	0.98	1.66
	Ganglionic	81.4 ± 4.5	82.4 ± 4.3	82.4 ± 4.3	0.95	1.94
	Postganglionic	64.4 ± 2.8	65.0 ± 2.3	64.7 ± 2.4	0.95	1.48

This table shows mean values ± standard deviations (SDs) for T2 values measured within S1, L5, and L4 (left- and right-sided T2 measurements collapsed). Measurements in these nerves were performed at three segments (preganglionic, ganglionic, postganglionic) and in three scans per subject by observer 1. Intraclass correlation coefficients (ICCs) and coefficients of variation (CVs) are presented for the different nerves and segments.

**Table 2:** Interobserver reliability

		Observer 1	Observer 2	ICC	CV (in %)
*S1* (mean T2 ± SD, in ms)	Preganglionic	74.3 ± 6.5	75.4 ± 5.3	0.78	4.81
	Ganglionic	86.0 ± 5.7	86.3 ± 4.3	0.77	3.18
	Postganglionic	64.5 ± 3.3	64.4 ± 4.8	0.60	4.76
*L5* (mean T2 ± SD, in ms)	Preganglionic	75.1 ± 5.5	72.8 ± 4.8	0.58	5.39
	Ganglionic	84.1 ± 4.0	83.8 ± 6.6	0.50	5.09
	Postganglionic	66.2 ± 3.6	63.2 ± 2.9	0.44	4.99
*L4* (mean T2 ± SD, in ms)	Preganglionic	72.6 ± 4.6	75.3 ± 8.7	0.22	8.89
	Ganglionic	81.4 ± 4.5	83.3 ± 7.6	0.63	5.52
	Postganglionic	64.4 ± 2.8	63.1 ± 3.6	0.18	5.11

This table depicts mean values ± standard deviations (SDs) for T2 values measured within S1, L5, and L4 (left- and right-sided T2 measurements collapsed). Measurements in these nerves were performed at three segments (preganglionic, ganglionic, postganglionic) in the first out of three scans per subject by two independent observers (observer 1 and observer 2). Intraclass correlation coefficients (ICCs) and coefficients of variation (CVs) are presented for the different nerves and segments.

**Fig. 3:**
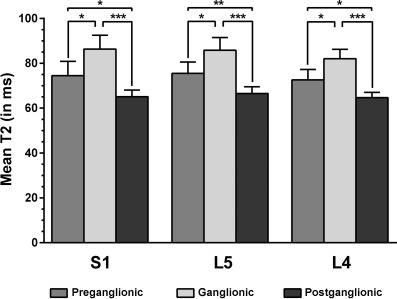
This figure plots the mean values ± standard deviations (SDs) for T2 values (in ms) of S1, L5, and L4 nerves. These values are derived from evaluation of three scans per subject by observer 1 in 15 healthy subjects (right-sided and left-sided T2 measurements and measurements of the three scans collapsed). The values are depicted separately for preganglionic, ganglionic, and postganglionic segments, and statistically significant differences in mean T2 values between these segments are shown (^*^p < 0.05, ^**^p < 0.01, ^***^p < 0.0001)

When averaging the T2 values derived from three scans measured by observer 1, S1 showed values of 74.5 ± 6.4 ms (preganglionic; range: 64.0–82.5 ms), 86.3 ± 6.2 ms (ganglionic; range: 77.7–97.6 ms), and 65.0 ± 3.0 ms (postganglionic; range: 59.6–71.0 ms), with statistically significant differences between these measurements (preganglionic vs. ganglionic: *p* = 0.0240; pre- vs. postganglionic: *p* = 0.0320, ganglionic vs. postganglionic: *p* < 0.0001; Fig. 3). For L5, T2 values of 75.5 ± 5.1 ms (preganglionic; range: 69.2–87.2 ms), 85.8 ± 5.6 ms (ganglionic; range: 78.0–99.8 ms), and 66.5 ± 3.0 ms (postganglionic; range: 61.0–72.0 ms) were revealed, and statistically significant differences between measurements were observed (preganglionic vs. ganglionic: *p* = 0.0346; pre- vs. postganglionic: *p* = 0.0072, ganglionic vs. postganglionic: *p* < 0.0001; Fig. 3). Moreover, T2 values for L4 were 72.5 ± 4.7 ms (preganglionic; range: 65.0–81.0 ms), 82.0 ± 4.2 ms (ganglionic; range: 76.6–92.8 ms), and 64.7 ± 2.4 ms (postganglionic; range: 60.8–69.6 ms), with statistically significant differences between these T2 values (preganglionic vs. ganglionic: *p* = 0.0152; pre- vs. postganglionic: *p* = 0.0191, ganglionic vs. postganglionic: *p* < 0.0001; Fig. 3).

## Discussion

The LSP area is commonly regarded as challenging in terms of MRN due to the need for high isotropic resolution to depict the complex oblique geometry of the nerves. It has been demonstrated recently that 3D TSE or 3D FSE combined with iMSDE preparation can achieve this high isotropic resolution and even resolve small nerve branches [2–6]; however, high-resolution 3D MRN of the LSP area can be significantly affected by B0 and B1 inhomogeneities that frequently occur in this region. The sequence used for T2 mapping of LSP nerves in this study is similar to a recently developed sequence that was successfully applied to T2 mapping of thigh muscles in healthy subjects [8]. When comparing the new T2-prepared 3D TSE sequence with standard multi-echo spin echo (MESE) sequences, a dependency of T2 values of MESE sequences on the transmit B1 field was shown, whereas the T2-prepared 3D TSE sequence was clearly less affected by the transmit B1 field [8]. Consequently, the used T2-prepared 3D TSE sequence enables B1-insensitive T2 quantification, thus tackling the issue of B1 sensitivity of T2 mapping, which is known to affect robust quantitative T2 measurements especially at 3 T (supplementary material).

The B1-insensitive T2-prepared 3D TSE sequence, combined with the presented approach of placing manual ROIs in reformatted images, allowed systematic measurements of T2 values of different LSP nerves and their segments. Although a limited amount of values had to be excluded due to measurements in areas with presumably high fractions of CSF or surrounding vessels (T2 >100 ms; observer 1: 7.8% excluded, observer 2: 11.9% excluded), the dataset analyzed remains considerably large, and the potential usefulness of our approach is reflected by excellent reproducibility. In this context, ICCs of at least 0.89 for all analyzed nerves and segments were observed (Table 1). Concerning the evaluation of interobserver reliability, the obtained ICCs were less convincing when comparing a more experienced to a relatively unexperienced observer, but still indicated fair to excellent agreement except for the L4 measurements (Table 2); however, the CVs calculated were considerably low, which was even true for the L4 (Table 2). The discrepancy between reproducibility and interobserver ICCs might be explained by potential difficulties in identification of anatomical structures during ROI placement since we compared a more experienced to a novice investigator. Moreover, manual placement of ROIs principally harbors the risk that two observers do not perform entirely equally, and small differences in placement may already lead to differences between investigators in a complex region such as the LSP.

In addition to evaluations of reproducibility and interobserver reliability, this study provides normative data on T2 values of three nerves of the LSP, separated into different segments (Table 1 and 2; Fig. 3). In this context, the pattern of values observed for these structures is comparable to a previous investigation using in-plane imaging, although the mean T2 measurements seem to be slightly higher in the present study [12]; however, literature on extraction of quantitative measures of LSP nerves is still limited, and application of MRN in this region is predominantly restricted to qualitative imaging without routine extraction of quantitative parameters. Regarding quantitative approaches, diffusion imaging has been used to outline normative values and microstructural organization of LSP nerves [12–14]. Furthermore, an iMSDE-prepared sequence for 3D imaging has been used to measure sciatic and femoral nerve angles and diameters in healthy subjects, and the obtained values might serve as normative data for comparison to patients with pathologies affecting the LSP [6].

From a clinical perspective, normative data derived from such 3D MRN may be used for identification and quantification of pathologies of LSP nerves. Frequent pathologies for which quantitative measurements could be regarded as helpful during diagnostics and monitoring might be demyelinating polyneuropathies or structural abnormalities leading to compression of nerves, such as disc protrusion or spinal stenosis [6, 15, 16]. Additionally, adding quantitative measurements to mere nerve visualization during high-resolution MRN might further enhance guidance of interventional nerve blocks and perineural injections. So far, promising results have already been achieved for qualitative MRN, but investigations on combined qualitative and quantitative high-resolution 3D MRN for this purpose have not yet been carried out [17].

Although our study adds valuable data to the limited amount of literature on high-resolution 3D MRN of LSP nerves, we have to bear some limitations in mind. First, no combination with diffusion imaging was established, which might have enhanced correct identification of nerves and accuracy, as suggested by investigations on peripheral nerve pathologies [18]. Second, we only enrolled healthy subjects in this study to establish a new quantitative MRN approach and to provide normative T2 values for clinical purposes; therefore, it has to be evaluated in further studies whether patients with lesions of LSP nerves show significantly altered measurements when compared to data derived from healthy subjects. Third, measurements leading to T2 values >100 ms were systematically removed from the dataset, but it is not clear whether definition of a rather arbitrary threshold is justifiable in later clinical use. Although only a limited fraction of measurements was removed due to this threshold, further studies are needed to refine the approach presented in this work; however, all removed values belonged to measurements in preganglionic or ganglionic segments while none of the postganglionic measurements had to be excluded. When our approach is applied among patients in future studies to evaluate differences in T2 mapping between the affected and non-affected side (e.g. nerves affected by compression or inflammation), postganglionic measurements that are performed distant to the spinal cord and, thus, distant to prominent CSF contamination might already be sufficient for clinical diagnosis. Fourth, partial volume effects may still be present in the manual drawing of the ROIs in the axial reformats; however, the present analysis with the reformatting of the 3D isotropic data should reduce partial volume effects compared to previous quantitative LSP imaging works, which have been traditionally based on straight axial two-dimensional imaging. Fifth, alterations of the T2 values within peripheral nerves could also in part be induced by the dependency of the T2-weighted signal intensity on the orientation of the nerve relative to the main magnetic field (so-called magic angle effect) [19]; however, the elevation of T2 values due to this effect should be small when the axis of a respective nerve forms a small angle with the main magnetic field [20]. This should be the case for the investigated nerve segments close to the spinal cord; however, the magic angle effect should be considered in further analysis. Finally, the present T2 preparation did not use flow or diffusion encoding gradients, thus giving rise to potential signal contamination by surrounding CSF and vessels. The T2 preparation modules with flow or diffusion encoding gradients could be used in the future to remove CSF and vessel signal contamination [5, 7, 21].

## Conclusion

The combination of 3D TSE imaging and adiabatic T2 preparation (T2-prepared 3D TSE) enables B1-insensitive and isotropic high-resolution T2 mapping of LSP nerves. This approach allowed measurements of the spatial variation of T2 values of different nerves (S1, L5, and L4) within different segments (preganglionic, ganglionic, and postganglionic) in healthy subjects, thus providing normative data with excellent reproducibility. The T2 mapping approach using T2-prepared 3D TSE might reflect a robust method for future diagnostics of LSP pathologies.

## Caption Electronic Supplementary Material

Characteristics of the T2-prepared 3D TSE sequence

## Abbreviations

3D: Three-dimensional
BIR-4: B1-insensitive rotation
CSF: Cerebrospinal fluid
CV: Coefficient of variation
FOV: Field of view
FSE: Fast spin echo
ICC: Intraclass correlation coefficient
iMSDE: Improved motion sensitized driven equilibrium
LSP: Lumbosacral plexus
MESE: Multi-echo spin echo
MIP: Maximum intensity projection
MRN: Magnetic resonance neurography
ROI: Region of interest
SPAIR: Spectral attenuated inversion recovery
SD: Standard deviation
TE: Echo time
TR: Repetition time
TSE: Turbo spin echo
VAPRO: Variable projection
