# Supplementary material for: High Isotropic Resolution T2 Mapping of the Lumbosacral Plexus with T2-Prepared 3D Turbo Spin Echo
Source: Clin Neuroradiol. 2018 Jan 10;29(2):223–30. doi: 10.1007/s00062-017-0658-9 (PMC6579865; doi:10.1007/s00062-017-0658-9)
Supplement: Supplementary file 1 — Characteristics of the T2-prepared 3D TSE sequence [file 62_2017_658_MOESM1_ESM.docx]

**Electronic Supplementary Material**

In this work a T2-prepared 3D TSE sequence was used according to the sequence diagram in Figure S1a. A modified BIR-4 pulse was implemented and gaps were introduced to obtain T2 preparation modules with different T2 weightings. Due to the nature of the adiabatic pulse, the sensitivity of this T2 preparation module to B1 inhomogeneity was reduced.

In Figure S1b, a histogram of the expected B1 and B0 inhomogeneities within the LSP is shown. The underlying B1 map was acquired with the dual TR method using a 3D gradient echo sequence with the following parameters: FOV = 38×38×8 cm^3^, acquired voxel size = 3.4×4.4×8 mm^3^, TR1/TR2/TE = 20/100/2.3 ms, flip angle = 60°. The B0 map was measured based on a 2-echo 3D gradient echo sequence with the following parameters: FOV = 38×38×8 cm^3^, acquired voxel size = 1.5×2×8 mm^3^, TR/TE1/ΔTE = 4.1/1.34/2.6 ms, flip-angle = 10° and two acquired echoes.

In Figure S1c, the sensitivity of the applied T2 mapping sequence to B1 and B0 errors was simulated with a Bloch simulation. The simulated parameters are as follows: T1/T2: 1200/70 ms, duration of T2 preparation = 20/40/60/80 ms, B1 = 13.5 µT, BIR-4 duration = 10 ms and BIR-4 frequency sweep = 3700 Hz.

Figure S1b shows a wide range of the B1 and B0 errors observed in vivo in the LSP area. However, the simulated T2 values shown in Figure S1c are stable over most of the possible B1 and B0 errors observed in vivo. The applied sequence can be therefore regarded as B1- and B0-insensitive in measuring T2 values in the LSP at 3 Tesla.

**
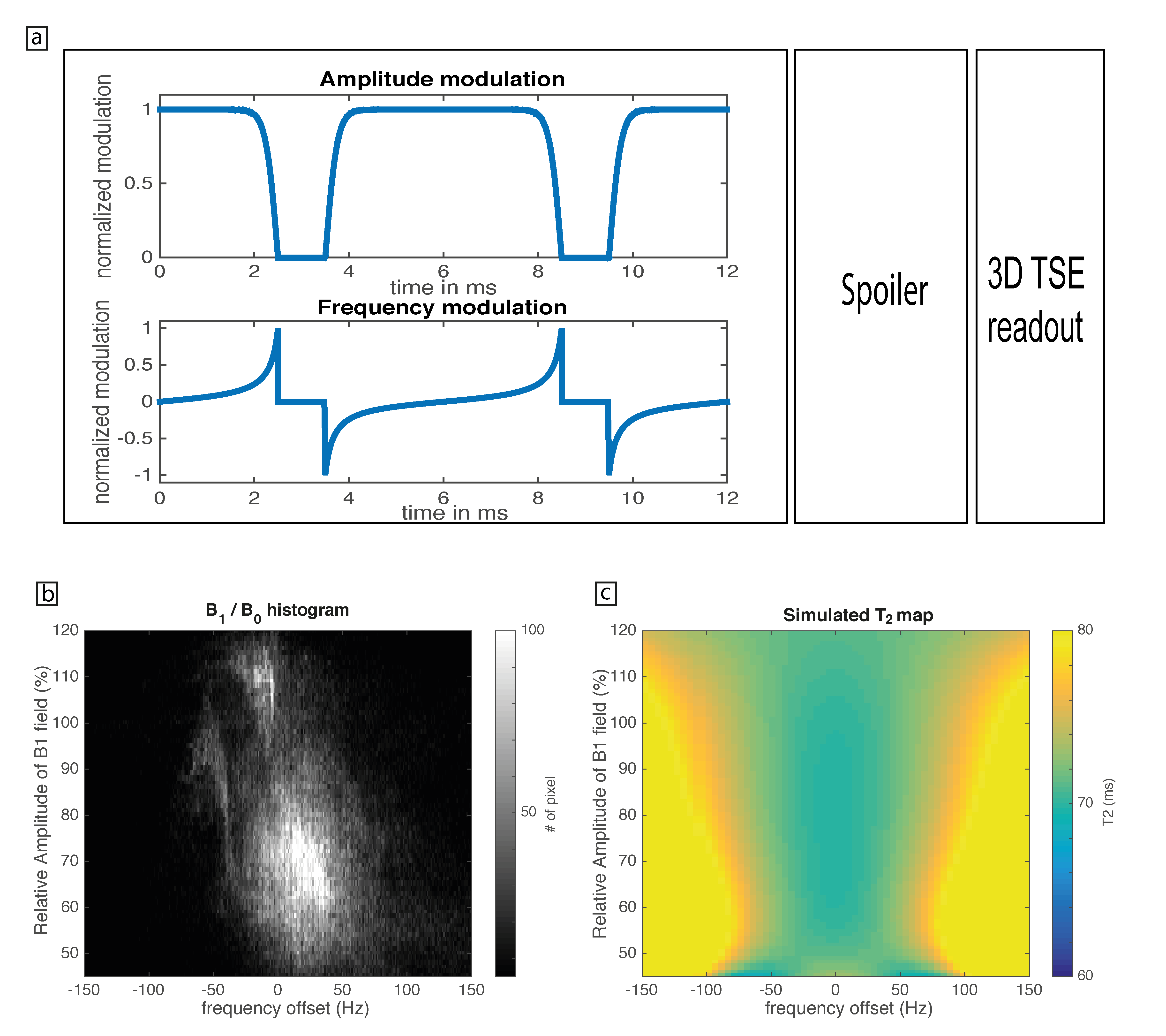
**

**Figure S1:** (a) Pulse sequence diagram of the employed T2-prepared 3D TSE showing the amplitude modulation and the frequency modulation of the T2 preparation module, the spoiler gradient, and the 3D TSE readout. (b) A histogram of the B1 and B0 errors present in in-vivo scanning of the LSP area. (c) Bloch simulation of expected T2 values for the same range of B1 and B0 errors as in (b). The simulated T2 values in (c) are stable over the range of the measured B1 and B0 inhomogeneities shown in (b).
